# Essential EU mobile workers in the Dutch food supply chain: occupational safety and health policies during and after the Covid-19 pandemic

**DOI:** 10.1093/eurpub/ckac129.406

**Published:** 2022-10-25

**Authors:** T deLange, S Mantu, N Skowronek, L Berntsen

**Affiliations:** 1 Faculty of Law, Radboud University, Nijmegen, Netherlands; 2 Research Institute for Dutch Labour Movement, De Burcht Institute, Amsterdam, Netherlands

## Abstract

The pandemic has revealed the dependence of many labour markets on EU mobile ((im)migrant) workers, particularly in sectors designated as essential for national economies. Despite many EU workers falling under the label of ‘essential workers’, they are nonetheless vulnerable to unhealthy and unfair working and living conditions linked to the type of work they perform (manual), the conditions of their employment (highly flexible) and their lack of social networks that makes them dependent on their employers for housing and health. Taking EU mobile workers in the meat and distribution sectors, two specific Food Supply Chain sectors in the Netherlands, as a case study, this contribution discusses how in the Netherlands protection during the Covid-19 pandemic was organized for this group of workers in relation to protective measures at the work place, quarantining, pay during periods of quarantine and protection against job loss, and access to public health services (testing, vaccines), topics that generated a great deal of insecurity among EU mobile workers. The analysis is based on data provided by a survey among 153 EU mobile workers (May-July 2021), 35 interviews with Polish and Romanian workers, and 50 interviews with national stakeholders. The aim is to problematize the disjuncture revealed by the pandemic between an EU legal regime that prioritizes mobility and national arrangements around welfare and social protection that leave many mobile workers vulnerable. We argue that EU free movement of workers needs to be flanked by measures to ensure that the people who move do so under decent and fair conditions.

